# Glucose-to-Lymphocyte Ratio (GLR) as a Predictor of Preoperative Central Lymph Node Metastasis in Papillary Thyroid Cancer Patients With Type 2 Diabetes Mellitus and Construction of the Nomogram

**DOI:** 10.3389/fendo.2022.829009

**Published:** 2022-04-26

**Authors:** Lingli Jin, Danni Zheng, Danni Mo, Yaoyao Guan, Jialiang Wen, Xiaohua Zhang, Chengze Chen

**Affiliations:** ^1^ Department of Breast Surgery, The First Affiliated Hospital of Wenzhou Medical University, Wenzhou, China; ^2^ Department of Thyroid Surgery, The First Affiliated Hospital of Wenzhou Medical University, Wenzhou, China; ^3^ Department of Plastic Surgery, Sir Run-Run Hospital Affiliated to Zhejiang University, Hangzhou, China

**Keywords:** GLR, PTC, nomogram, NRI, IDI, DCA

## Abstract

**Background:**

Detection of metastasis of central lymph nodes in papillary thyroid cancer is difficult before surgery. The role of routine or preventive central lymph node dissection in the management of papillary thyroid cancer remains inconclusive. Moreover, glucose metabolism and systemic inflammation are related to the aggressiveness of several malignant tumors and the prognoses of these patients. This study aimed to construct a nomogram based on the readily available preoperative clinical features for predicting the occurrence of preoperative central lymph node metastasis in patients with papillary thyroid cancer and type 2 diabetes mellitus. The findings may underlie clinical implications for determining the appropriate treatment strategies for these patients.

**Methods:**

A total of 419 patients were enrolled. We used the receiver operating characteristic curves to determine the best cut-off value and converted the continuous into categorical variables. Next, a single-factor logistic analysis for the independent variables was performed, following which a multivariate regression analysis was conducted for the selected significant risk factors. Finally, the nomogram was constructed and verified using external data; the existing data were compared with the original model.

**Results:**

According to the receiver operating characteristic curves, the best cut-off values ​​for glucose-to-lymphocyte ratio and tumor size were 4.23 cm and 0.95 cm, respectively. Findings from the multivariate logistic regression analysis suggested that age, bilateral tumors, maximum tumor size, and the ratio of glucose-to-lymphocytes were independent risk factors for preoperative central lymph node metastasis. The C-indexes in the training and the external validation data sets were 0.733 and 0.664, respectively. Both calibration curves and the Hosmer-Lemeshow tests indicated that the model was well-calibrated. Through decision curve analysis, the predictive model was estimated to have strong clinical applicability and greater benefits. To compare the performance of the new with that of the original model, we performed a net reclassification index and the integrated discrimination improvement analyses, both of which indicated that the new model had a better predictive ability.

**Conclusion:**

In patients with type 2 diabetes mellitus and papillary thyroid cancer, a high preoperative glucose-to-lymphocyte ratio was an independent predictor of the preoperative central lymph node metastasis. The nomogram so constructed could better predict the preoperative central lymph node metastasis in these patients.

## Introduction

Diabetes is a common chronic metabolic disease, with annually increasing morbidity and mortality rates (1). Diabetes and cancer are closely related in that diabetes can cause an increase in the death rate among cancer patients (2–4). Previously, two large-scale studies have shown that a history of diabetes may be related to the occurrence of thyroid cancer (5, 6). Patients with diabetes mellitus (DM) are at a higher risk of developing thyroid cancer (7–9); relative to those without DM, the incidence of thyroid cancer in patients with DM increases by 20% (8). People with DM and elevated fasting blood sugar levels are more likely to develop thyroid cancer, thereby indicating a positive correlation. Consequently, elevated blood sugar may affect the survival time and clinical outcomes in cancer patients (10–12). Previous studies also show that the development of cancer increases the risk of diabetes (13). The concentrations of inflammatory cytokines, such as TNF-a, CRP, and IL-1 in diabetic patients are substantially higher relative to non-diabetic patients (14–17). In addition, it is generally believed that chronic inflammation is indispensable in the occurrence and development of diabetes, along with the pathogenesis of its complications (18). Different cell types secrete several inflammatory cytokines that are released into the blood circulation and produce differential effects on varying tissue types (19–21).

In several human cancer types, recently, systemic inflammation and the tumor microenvironment play indispensable roles in activating tumor cell proliferation, invasion, and metastases (17, 21–24). Moreover, the peripheral blood parameters of these markers, including white blood cells, lymphocytes, neutrophils, platelet count, and monocytes, are cheap and can be easily obtained to further evaluate the prognoses of various human cancers. Several studies on hematological indicators have been published, some of which show that glucose-to-lymphocyte (GLR) is a reliable prognostic marker for pancreatic (25) and gallbladder cancers (26). However, there are no published reports on the association of the risk of preoperative central lymph node metastasis (CLNM) and GLR in papillary thyroid cancer (PTC) patients with type 2 DM (T2DM). Surgical resection is the most effective treatment for PTC but the role of prophylactic central lymph node dissection (CLND) in PTC remains controversial (27, 28), and the patients with CLND experience higher rates of postoperative hypocalcemia (27, 29). Therefore, identification of an effective way to exclude low-risk patients from CLND is necessary. The purpose of this study was to construct the nomogram for improving the accuracy of predicting the possibility of lymph node metastasis in the central region before surgery using easily accessible and inexpensive hematological indicators.

## Patients and Methods

### Patient Recruitment

A retrospective analysis was performed for a total of 419 patients with PTC and T2DM who underwent thyroid lobectomy and lymph node dissection in two clinical centers; among them, 319 were enrolled from the First Affiliated Hospital of Wenzhou Medical University between 2017 and 2020, and 100 from the Zhejiang Provincial People’s Hospital between 2018 and 2020. The information on the clinical pathology of patients was collected. None of the patients showed central or lateral lymph node enlargement, assessed by preoperative imaging examinations or biopsy scans.

The exclusion criteria were as follows:

1) Incomplete clinicopathological characteristics.
2) Patients whose pathology suggested the presence of malignant PTC without lymph node dissection.
3) Patients with poor blood sugar control after admission; the standard range for poor blood glucose control was hBA1c> 7% and those who could not be operated upon.
4) Patients with hyperthyroidism, history of thyroid radiation, or thyroid surgery.
5) The preoperative imaging examinations, using the results of the preoperative ultrasound, CT, MRI, or biopsy scans, suggested the presence of swollen lymph nodes in the central and lateral areas.
6) Patients with other malignancies.
7) Patients with a history of infections or other inflammation (excluding Hashimoto’s thyroiditis).

### Data Acquisition

All the selected patients underwent a comprehensive preoperative evaluation, including thyroid ultrasound, blood indicator assessment, and thyroid function tests. The basic information, including age, gender, height and weight, and diabetes-related medical history (including medications for the treatment of diabetes) were obtained.

Thyroid ultrasound and cervical CT examinations were used to assess the maximum tumor size, lateral position, multifocal, and chronic lymphocytic thyroid inflammation. The results of intraoperative rapid freezing and postoperative paraffin pathology were also analyzed. Hematology indicators were typically collected within a week before the operation. The formula for GLR calculation was as follows: GLR=glucose count/lymphocyte count.

### Statistical Analysis

We used the ROC curves to determine the best cut-off values for the variables and stratified them. Using the chi-square test, we evaluated if the two sets of data were comparable. Single- and multi-factor logistic analyses were used to identify the factors that were significantly related to preoperative CLNM and the predictive model was constructed. We calculated the area under the ROC curve (AUROC) for the training and the validation sets. The calibration curve was used to evaluate the agreement between actual observations and predicted values, ​​and further, visualize these results. The Hosmer-Lemeshow test was used to assess the significance of the calibration curves (p>0.1 indicated good consistency). The DCA curves were used to measure the net benefits for different threshold probabilities, thereby aiding the determination of the clinical benefits of using the nomogram in clinical settings. The NRI and IDI values were used to quantitatively analyze and evaluate the improvements in the diagnostic performance of the new relative to the original model. NRI>0 suggested improvement; NRI<0 indicated negative improvement, while NRI=0 showed no improvement. IDI>0 implied improvement; IDI<0 was a negative improvement, and IDI=0 indicated no improvement. All the statistical analyses were performed using R (version 4.0.2) and SPSS 25.0 software. P < 0.05 was considered statistically significant.

## Results

### Clinical Characteristics and Basic Information

In this study, we included 419 confirmed cases of PTC and T2DM from the above-mentioned two hospitals. All the data were divided into two based on their source as the training (n=319, 76%) and external test (n=100, 24%) data sets. For objectivity, ROC analysis was used to select the best cut-off value for each continuous variable. The results of the chi-square analysis, as shown in **Table 1**, indicated that the two data sets were not significantly different (P>0.5), and thus, were consistent and comparable.

**Table 1:** Baseline characteristics of patients in the training and external validation data sets.

Variables	Training Data Set N=319	Validation Data Set N=100	P-value
Age			
>55Y	181	51	0.314
≤55Y	138	49	
Gender			
Female	211	68	0.731
Male	108	32	
Medicine for diabetes			
Metformin(-)	173	62	0.172
Metformin(+)	146	38	
BMI(kg/m2)			
>32.49	3	3	0.13
≤32.49	316	97	
Maximum diameter of mass			
>9.5mm	107	34	0.933
≤9.5mm	212	66	
Multifocality			
Solitary	251	73	0.236
Multiple	68	27	
CLNM			
CLNM(-)	185	55	0.597
CLNM(+)	134	45	
Laterality			
Unilateral	289	89	0.639
Bilateral	30	11	
Hashimoto’s thyroiditis			
Absent	283	89	0.937
Present	36	11	
TC(mmol/L)			
>7.235	14	5	0.798
≤7.235	305	95	
TG(mmol/L)			
>3.975	36	16	0.212
≤3.975	283	84	
glucose(mmol/L)			
>7.25	190	60	0.938
≤7.25	129	40	
Albumin(g/L)			
>42.15	212	68	0.775
≤42.15	107	32	
AGR			
>1.25	260	82	0.911
≤1.25	59	18	
neutrophil(x10^9)			
>4.395	101	32	0.949
≤4.395	218	68	
mononuclear(x10^9)			
>0.355	207	68	0.568
≤0.355	112	32	
lymphocyte(x10^9)			
>1.435	275	80	0.132
≤1.435	44	20	
lymphocytepercent(%)			
>37.35	70	25	0.524
≤37.35	249	75	
TSH(mIU/L)			
>0.855	255	82	0.65
≤0.855	64	18	
GLR			
>4.23	161	47	0.545
≤4.23	158	53	

### Association Between Clinical Features and GLR in the Two Cohorts

The best cut-off value for GLR was 4.23. Therefore, we divided the patients into two according to their GLR values as the low GLR level group (≤4.23), comprising 211 patients (50.4%) and the high GLR level group (>4.23), comprising 208 cases (49.6%). As listed in **Table 2**, the GLR levels were significantly related to age (P=0.004), metformin consumption (P=0.026), blood sugar (P<0.001), lymphocyte count (P<0.001), and lymphocyte percentage (P<0.001). The higher GLR levels were associated with older age, no metformin consumption, high blood sugar, higher lymphocyte counts, and lower lymphocyte percentage.

**Table 2:** Correlations between GLR and the clinical characteristics of patients in all datasets.

Variables	GLR ≤ 4.23N=211	GLR>4.23N=208	P-value
Age			
>55Y	102	130	0.004
≤55Y	109	78	
Gender			
Female	141	138	0.917
Male	70	70	
Medicine for diabetes			
Metformin(-)	107	128	0.026
Metformin(+)	104	80	
BMI(kg/m2)			
>32.49	4	2	0.421
≤32.49	207	206	
Maximum diameter of mass			
>9.5mm	73	68	0.68
≤9.5mm	138	140	
Multifocality			
Solitary	162	162	0.787
Multiple	49	46	
CLNM			
CLNM(-)	126	114	0.31
CLNM(+)	85	94	
Laterality			
Unilateral	189	189	0.656
Bilateral	22	19	
Hashimoto’s thyroiditis			
Absent	185	187	0.47
Present	26	21	
TC(mmol/L)			
>7.235	9	10	0.79
≤7.235	202	198	
TG(mmol/L)			
>3.975	23	29	0.345
≤3.975	188	179	
glucose(mmol/L)			
>7.25	66	184	<0.001
≤7.25	145	24	
Albumin(g/L)			
>42.15	145	135	0.407
≤42.15	66	73	
AGR			
>1.25	168	174	0.287
≤1.25	43	34	
neutrophil(x10^9)			
>4.395	61	72	0.21
≤4.395	150	136	
mononuclear(x10^9)			
>0.355	151	124	0.1
≤0.355	60	84	
lymphocyte(x10^9)			
>1.435	202	153	<0.001
≤1.435	9	55	
lymphocytepercent(%)			
>37.35	73	22	<0.001
≤37.35	138	186	
TSH(mIU/L)			
>0.855	172	165	0.572
≤0.855	39	43	

### Univariate and Multivariate Analyses for Preoperative CLNM Variables

In univariate analysis, age (P=0.054), maximum tumor diameter (P<0.001), tumor bilaterality (P<0.001), and GLR (P=0.063) were found to be significantly associated with preoperative CLNM (**Table 3**). Further, a multivariate logistic regression analysis was performed to identify the important variables that were significantly related to preoperative CLNM. The results showed that age (≤55 years, P=0.019), maximum tumor diameter (>0.95cm, P<0.001), bilateral tumor (P<0.001), and high GLR (P=0.041) were independent risk factors for preoperative CLNM (**Table 3**).

**Table 3:** Univariate and multivariate analyses for logistic regression in the training data set.

Variables	Univariate Analysis of preoperative CLNM			Multivariate Analysis of preoperative CLNM		
	OR	95% CI	P-value	OR	95% CI	P-value
Age	0.581	0.334-1.009	0.054	0.0542	0.324-0.904	0.019
>55Y						
≤55Y						
Gender	0.917	0.506-1.662	0.774			
Female						
Male						
Medicine for diabetes	0.691	0.407-1.174	0.172			
Metformin(-)						
Metformin(+)						
BMI(kg/m2)	2.334	0.411-13.258	0.339			
>32.49						
≤32.49						
Maximum diameter of mass	4.195	2.403-7.323	<0.001	4.243	2.499-7.205	<0.001
>9.5mm						
≤9.5mm						
Multifocality	0.745	0.389-1.423	0.372			
Solitary						
Multiple						
Laterality	9.022	2.803-29.035	<0.001	8.115	2.603-25.301	<0.001
Unilateral						
Bilateral						
Hashimoto’s thyroiditis	1.391	0.615-3.148	0.428			
Absent						
Present						
TC(mmol/L)	1.846	0.501-6.801	0.357			
>7.235						
≤7.235						
TG(mmol/L)	1.361	0.577-3.208	0.481			
>3.975						
≤3.975						
Albumin(g/L)	1.278	0.701-2.329	0.423			
>42.15						
≤42.15						
AGR	1.682	0.794-3.563	0.175			
>1.25						
≤1.25						
neutrophil(x10^9)	1.581	0.874-2.859	1.13			
>4.395						
≤4.395						
mononuclear(x10^9)	1.291	0.692-2.409	0.422			
>0.355						
≤0.355						
TSH(mIU/L)	1.697	0.865-3.328	0.124			
>0.855						
≤0.855						
GLR	1.691	0.972-2.941	0.063	1.704	1.021-2.843	0.041
>4.23						
≤4.23						

### Construction of the Nomogram

**Figure 1:**
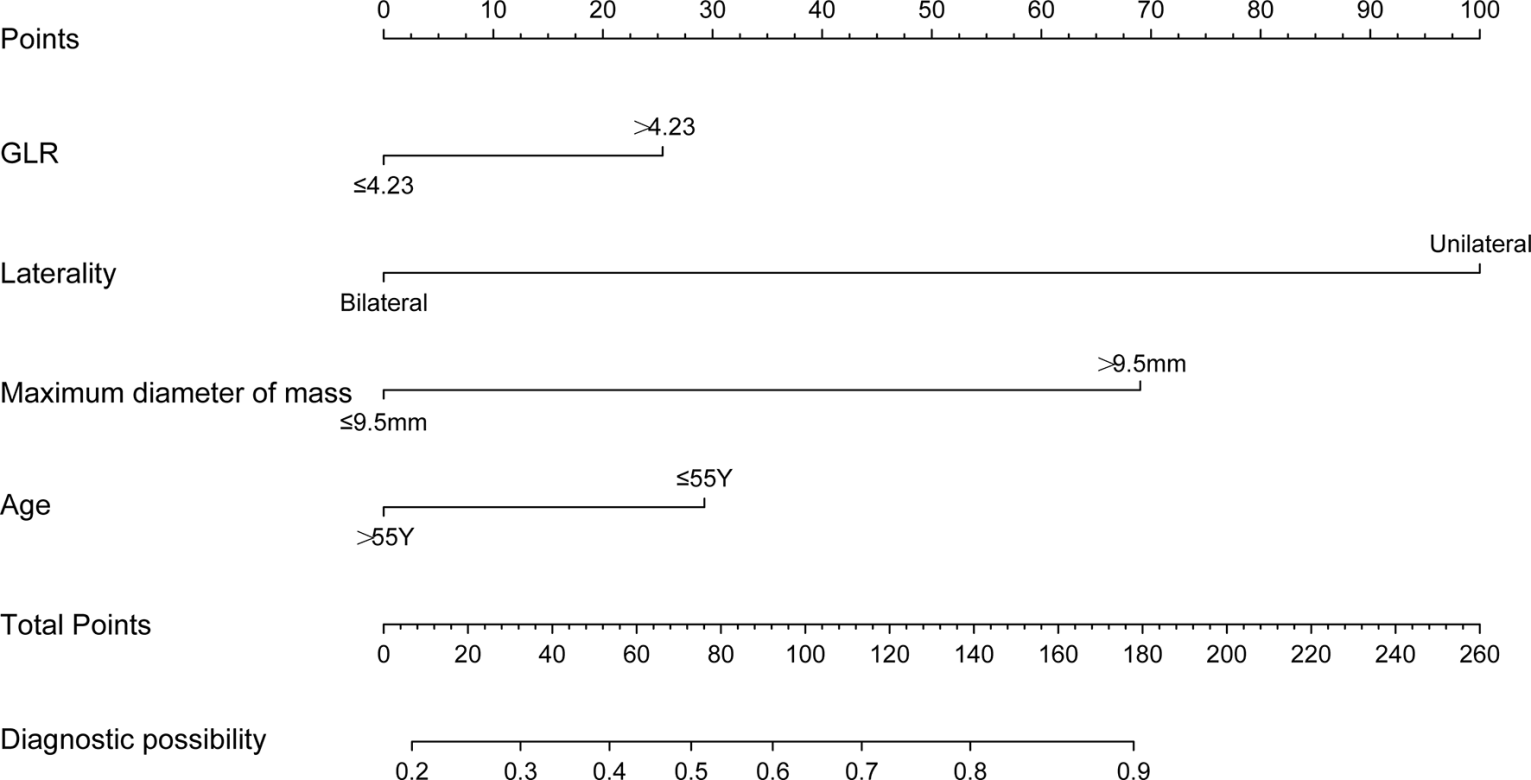
Nomogram for predicting the preoperative CLNM in T2DM-PTC patients.

The independent factors selected based on the results of the multivariate analysis were used to construct the nomogram for predicting the individual risk of preoperative CLNM (**Figure 1**). The scores for each independent predictor were plotted and serially summed up to obtain the total score for confirming the possibility of preoperative CLNM.

### Evaluation of the Nomogram

**Figure 2:**
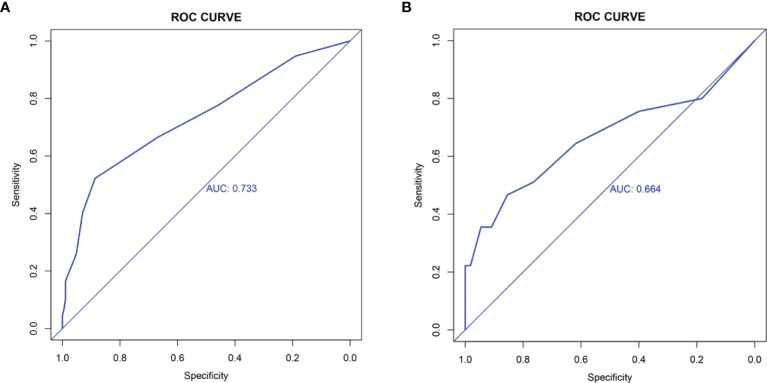
Receiver operating characteristics (ROC) analysis in **(A)** training data set; **(B)** external validation data set. The AUC for the nomogram in the training data set is 0.733, while for the external validation data set is 0.664.

**Figure 3:**
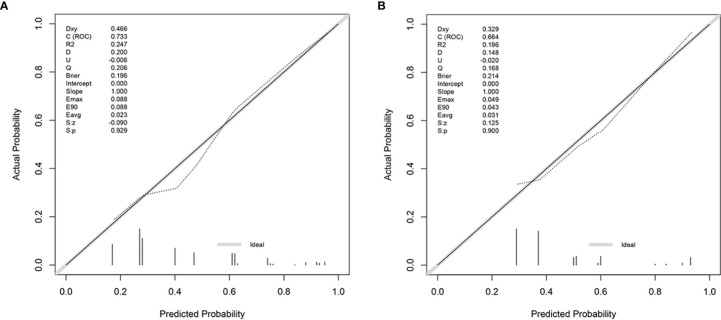
Calibration curves for the nomogram for **(A)** training data set and **(B)** external validation data set. The calibration curves show a high degree of consistency between the predicted value and the actual situation, as the p-values are 0.5416304 (>0.1) and 0.5803292 (>0.1), respectively from the Hosmer-Lemeshow test.

**Figure 4:**
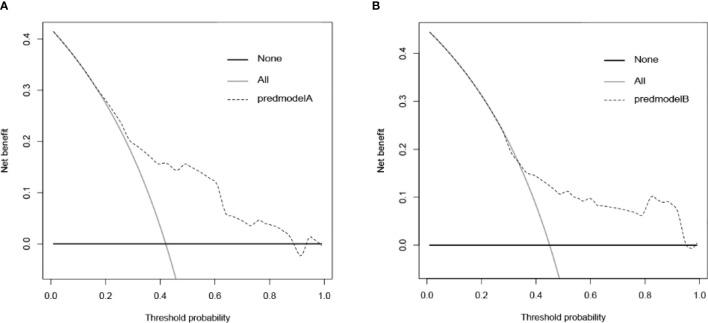
Decision curve analysis (DCA) for the nomogram in **(A)** training data set and **(B)** external validation data set. The training data are concentrated between approximately 20% and 90% and the external validation data are approximately between 30% and 90% of the total distribution.

To evaluate the ability of the nomogram for predicting the preoperative CLNM in PTC and comorbid T2DM patients, we used an R package to perform ROC analysis and obtained an AUROC of 0.733 in the internal training set (**Figure 2A**) and an AUROC of 0.664 in the external validation set (**Figure 2B**). The calibration curves indicated a high degree of consistency between the predicted value and the real situation, as shown in **Figures 3A, B** (by Hosmer-Lemeshow test, internal training set, p =0.5416304>0.1 and external validation set p=0.5803292>0.1). **Figures 4A, B** show the DCA diagrams for the internal and the external data sets, respectively. The dotted line in the figure represents the net benefit, while the gray line shows the net clinical benefit that these patients received (with the assumption that all patients had undergone treatment for CLND). The black color represents the net clinical benefit, assuming that all patients did not undergo the treatment for CLND. Through DCA, the net clinical benefit for patients who were predicted to and underwent selective CLND through this nomogram was found to be markedly higher than that for all patients who had undergone or not received treatment for CLND. The training data were concentrated between approximately 20% and 90% of the distribution, while the external validation data were between 30% and 90%.

### Comparison With the Original Model

**Figure 5:**
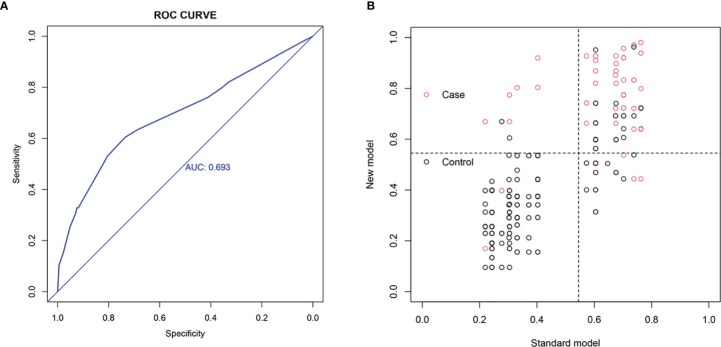
Comparison with the original model. **(A)** ROC analysis. **(B)** Net reclassification index (NRI). NRI=0.04171037>0, (IDI=0.0902>0) indicates that the diagnostic performance of the new model is improved relative to the original model.

To further verify that the nomogram indeed had better predictive ability and accuracy, we compared the new with the original model. Through literature review, we obtained a nomogram published by Andrew M. Thompson et al., in 2014, for the prediction of preoperative CLNM in PTC patients (30). This nomogram includes age, gender, tumor size, and tumor multifocality as the parameters. The AUC obtained after substituting our data into the original model was 0.693 (**Figure 5A**), lesser than 0.733, the value obtained using the new model. We used NRI and the IDI quantitative analyses to evaluate the improved diagnostic performance of the new model relative to the original model. As shown in **Figure 5B**, NRI=0.04171037>0, (IDI=0.0902>0) indicated that the diagnostic performance of the new model improved significantly relative to the original model.

## Discussion

At present, surgeons routinely perform preventive dissection of lymph nodes in the central region of the neck in radical thyroid cancer surgery for PTC patients (28) but preventive CLND can lead to an increased risk of postoperative hypocalcemia and other risks (27, 29). How to accurately identify patients who do not need preventive CLND before surgery needs to be addressed for effective clinical practice.

Many previous studies show that GLR can be used as a prognostic indicator for patients with pancreatic (25) and pT2 gallbladder cancers (26), and hospitalized patients with acute severe pancreatitis (31). However, there is no study on the role of GLR in PTC. Therefore, our important core hypothesis was as follows: preoperative GLR is significantly correlated with CLNM in PTC patients with T2DM.

In this study, we retrospectively analyzed the clinical data of 419 patients with PTC complicated by T2DM. Univariate and multivariate logistic regression analyses showed that age < 55 years, bilateral tumor, maximum tumor size > 9.5mm, and higher GLR ratio were all independent risk factors for CLNM. The inflammatory cytokines in diabetic patients are higher than those in non-diabetic patients (32). Systemic inflammation plays key roles in the occurrence, development, progression, metastasis, and recurrence of several solid tumors. Some documented literature suggests that lymphocytes can activate cell-mediated immune responses and tumor cell lysis (23, 24, 32). Thus, we speculated that GLR could predict the CLNM of PTC patients with T2DM by reflecting the levels of inflammation, wherein a high GLR value implied high blood glucose or low lymphocyte level. Since we excluded patients with hBA1c>7% or poor blood glucose control before surgery, high GLR may be more closely related to low lymphocyte levels. Lymphocytes are a major component of anti-tumor immunity, stimulating the release of cytokines such as interferons and TNF-α, thereby exerting protective effects (33). However, in our previous screening, no significant correlation was found between the number of lymphocytes or the blood glucose alone before surgery and CLNM in patients with PTC combined with T2DM. These results indicated that GLR was an independent significant new indicator for predicting preoperative CLNM in patients with PTC combined with T2DM. Relative to unilateral tumors, the incidence of CLNM in bilateral tumors increased by 8.115 times, consistent with previous studies (34). We speculated that this may be due to the more aggressive bilateral tumors. Moreover, results of both univariate and multivariate logistic regression analyses indicated that tumor > 9.5 mm was an independent risk factor for CLNM. The risk of CLNM increased 4.243-fold when the tumor diameter was greater than 9.5 mm. In addition, in both the training (≤ 9.5mm: > 9.5mm = 212:107) and the validation group (≤ 9.5mm: > 9.5mm = 66:34) the ratio, ≤ 9.5mm: > 9.5mm, was approximately 2:1. In the subgroup whereby the maximum diameter of mass was <9.5mm, the CLNM in the training group comprised 63 patients, and the CLNM in the external validation group of 23 patients, while in the subgroup whereby the maximum diameter of mass was >9.5mm, the CLNM in training group comprised 71 patients, and that in the external validation group of 22 patients. In a population more than twice the size of patient number whose maximum diameter of mass was > 9.5mm, there were similar proportions of CLNM cases. This further confirmed that in the patients whose maximum diameter of mass was <9.5mm, CLNM occurrence was difficult, consistent with previous literature (35–37). In terms of age, 55 years the recommended cut-off in the AJCC 8th edition thyroid cancer TNM stage (38), and less than 55 years was found to be an independent risk factor for CLNM, consistent with the literature (32, 35).

After confirming that high GLR was an independent risk factor for CLNM in PTC patients with T2DM, we further examined the clinical value of this new indicator for PTC patients with T2DM for improved prognoses, survival, treatment, and reduced surgical harm due to CLND. We further utilized GLR, unilateral and bilateral factors, tumor size, and age to construct a preoperative CLNM prediction model for the target population of PTC patients with T2DM. To verify that our model showed significantly improved CLNM for the target population of PTC patients with T2DM relative to the existing models, we compared our model with a known model. The preoperative CLNM prediction model described in Andrew M. Thompson et al., includes age, gender, tumor size, and tumor multifocality, and the target population was PTC patients (30). We input our data into the model of Andrew M. Thompson et al. and obtained an AUC of 0.693, smaller than our new model. This indicated that our model had a higher accuracy for distinguishing PTC patients with T2DM with or without CLNM. To further verify the good diagnostic efficiency of our model, we performed NRI and IDI quantitative analyses, and the results suggested that our new model including GLR and the excluding tumor multifocal and gender as factors, substantially improved the diagnostic efficacy relative to the model described by Andrew M. Thompson et al. Moreover, as compared to the existing models, the new model included fewer factors, calculating the risk scores more concise. Additionally, GLR is an easily obtained clinical indicator. Before each patient is admitted to the hospital for surgery, routine examinations for blood indicators, such as blood glucose and B-ultrasound are performed. Therefore, our model is universal and can be generalized. Without additional harm to patients or more tests, more meaningful clinical decisions and individualized treatment options can be taken in the future.

However, our study also has some limitations. The study has a retrospective design and some data bias may exist. The sample size was not large enough and more samples may be needed to further verify our model. We hope that in the future there will be more sensitive indicators to better predict CLNM and lymph node metastasis, to facilitate a solid theoretical basis for clinical decision making for the benefit of these patients.

In conclusion, GLR was found to be an independent risk factor for PTC and T2DM. Our proposed nomogram could be used to predict preoperative CLNM, which showed the benefits of good sensitivity and specificity.

## Data Availability Statement

The raw data supporting the conclusions of this article will be made available by the authors, without undue reservation.

## Ethics Statement

The studies involving human participants were reviewed and approved by the ethics committee of Zhejiang Provincial People’s Hospital and the ethics committee of First Affiliated Hospital of Wenzhou Medical University. The patients/participants provided their written informed consent to participate in this study

## Author Contributions

All the authors have made significant contributions to the study design, data acquisition, analysis, and interpretation. They have substantially participated in drafting the manuscript and critically revising the important content and assume responsibility for all aspects of this work. All authors contributed to the article and approved the submitted version.

## Funding

This study was supported by the Scientific Research Foundation of Wenzhou, Zhejiang Province, China (Y20210948).

## Conflict of Interest

The authors declare that the research was conducted in the absence of any commercial or financial relationships that could be construed as a potential conflict of interest.

## Publisher’s Note

All claims expressed in this article are solely those of the authors and do not necessarily represent those of their affiliated organizations, or those of the publisher, the editors and the reviewers. Any product that may be evaluated in this article, or claim that may be made by its manufacturer, is not guaranteed or endorsed by the publisher.
